# A Unique Presentation of an Upper Subscapular Nerve Variation Accompanied by an Accessory Subscapular Artery

**DOI:** 10.7759/cureus.35862

**Published:** 2023-03-07

**Authors:** Carley M Olson, Benjamin J Herstam, Yun Tan, Daniel T Daly

**Affiliations:** 1 Department of Surgery, Center for Anatomical Science and Education, Saint Louis University School of Medicine, St. Louis, USA; 2 Department of Physical Therapy, Doisy College of Health Sciences, Saint Louis University, St. Louis, USA

**Keywords:** brachial plexus, subclavian artery abnormal branching, upper subscapular nerve, subscapular artery, anatomical variation

## Abstract

The presence of an upper subscapular nerve branching from the posterior division of the superior trunk, and it being accompanied by an accessory subscapular artery, is of both clinical and surgical significance. During routine dissection of the root of the neck in a 75-year-old male cadaver, an unusual branch from the third part of the right subclavian artery was observed lateral to the dorsal scapular artery. Continued dissection revealed that this artery traveled between the anterior divisions of the superior and middle trunks of the brachial plexus before traveling alongside a nerve from the posterior division of the superior trunk of the brachial plexus. This artery and nerve descended on the anterior aspect of the subscapularis muscle before piercing into its muscle belly. We believe this to be a previously unreported unique variation of the upper subscapular nerve that is accompanied by an accessory subscapular artery on its course to the subscapularis muscle. Knowledge of anatomical variations like this may lead to decreased complications in nerve blocks and surgical procedures related to the shoulder.

## Introduction

The brachial plexus is a network of nerves that provides both somatic motor and sensory innervation to the upper extremity, including the rotator cuff. As the brachial plexus travels through the posterior triangle of the neck and then into the axilla, arm, forearm, and hand, it contains various regions that are named according to how the plexus is formed [1]. The most proximal portions of the brachial plexus, called roots, are the ventral primary rami of spinal nerves, including C5-C8 and T1 [2]. These roots combine in a way to form three trunks [3]. Trunks will proceed to divide into anterior and posterior divisions that coalesce into cords, named in relation to the second of three parts of the axillary artery, before giving rise to its terminal branches. While there are five terminal branches of the brachial plexus, many of the branches arise more proximally from the cords with a few branches emerging from roots and the upper trunk [2,3].

Typically, the upper subscapular nerve emerges from the posterior cord along with the lower subscapular nerve to innervate the subscapularis muscle, one of the four muscles making up the rotator cuff, which collectively assist in producing a wide range of shoulder movement while maintaining the stability of the glenohumeral joint [1,4].

The vascular supply to the rotator cuff muscles typically arises from multiple arteries, including the subscapular artery, posterior circumflex humeral artery, dorsal scapular artery, and suprascapular artery [4,5]. Both the subscapular and posterior circumflex humeral arteries originate from the axillary artery, and the suprascapular artery, which is usually a branch of the thyrocervical trunk. However, the dorsal scapular artery directly comes off the third part of the subclavian artery in about three out of four individuals [6].

The variation described in this case is a novel finding of unique neurovascular branching of the posterior division of the superior trunk and the third part of the subclavian artery, both of which terminate in the subscapularis muscle. The relations of the brachial plexus to surrounding muscles or blood vessels are very complex, and this complexity must be comprehensively understood by physicians engaged in surgical intervention in this region [7]. Awareness of this neurovascular variation is also important for radiologists and anesthesiologists due to clinical and surgical procedures in the axilla associated with clavicle fractures, shoulder dislocations, and supraclavicular nerve blocks.

## Case presentation

A 75-year-old male donor was received through the Saint Louis University (SLU) Gift of Body Program of the Center for Anatomical Science and Education (CASE) with signed informed consent from the donor. The donor’s self-reported medical history included Alzheimer’s disease and Parkinson’s disease. Every effort was made to follow all local and international ethical guidelines and laws that pertain to the use of human cadaveric donors in anatomical research [8]. The CASE Gift Body Program abides by all rules set forth by the Uniform Anatomical Gift Act.

**Figure 1:**
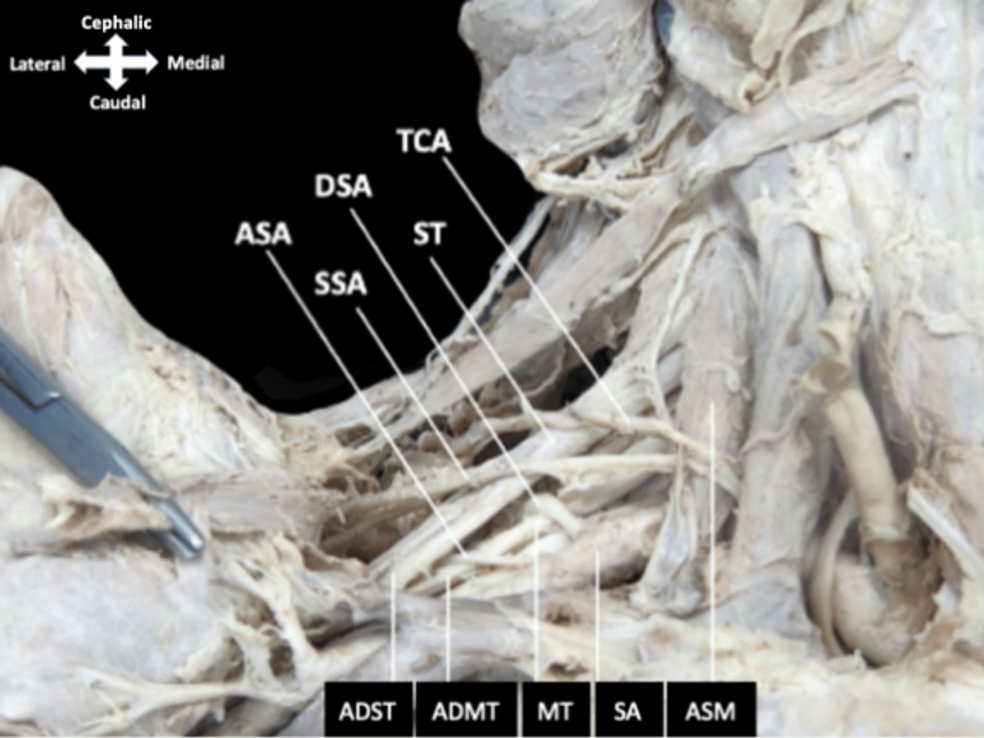
Root of the neck and posterior cervical triangle The transverse cervical artery and suprascapular artery course laterally across the anterior surface of the anterior scalene muscle. The third part of the subclavian artery can be seen lateral to the anterior scalene muscle where it gives off the dorsal scapular artery before it travels between the superior trunk and middle trunk. The accessory subscapular artery can be seen emerging from the subclavian artery lateral to the origin of the dorsal scapular artery before traveling between the anterior divisions of the superior and middle trunks. ADMT, anterior division of middle trunk; ADST, anterior division of superior trunk; ASA, accessory subscapular artery; ASM, anterior scalene muscle; DSA, dorsal scapular artery; MT, middle trunk; SA, subclavian artery; SSA, suprascapular artery; ST, superior trunk; TCA, transverse cervical artery.

**Figure 2:**
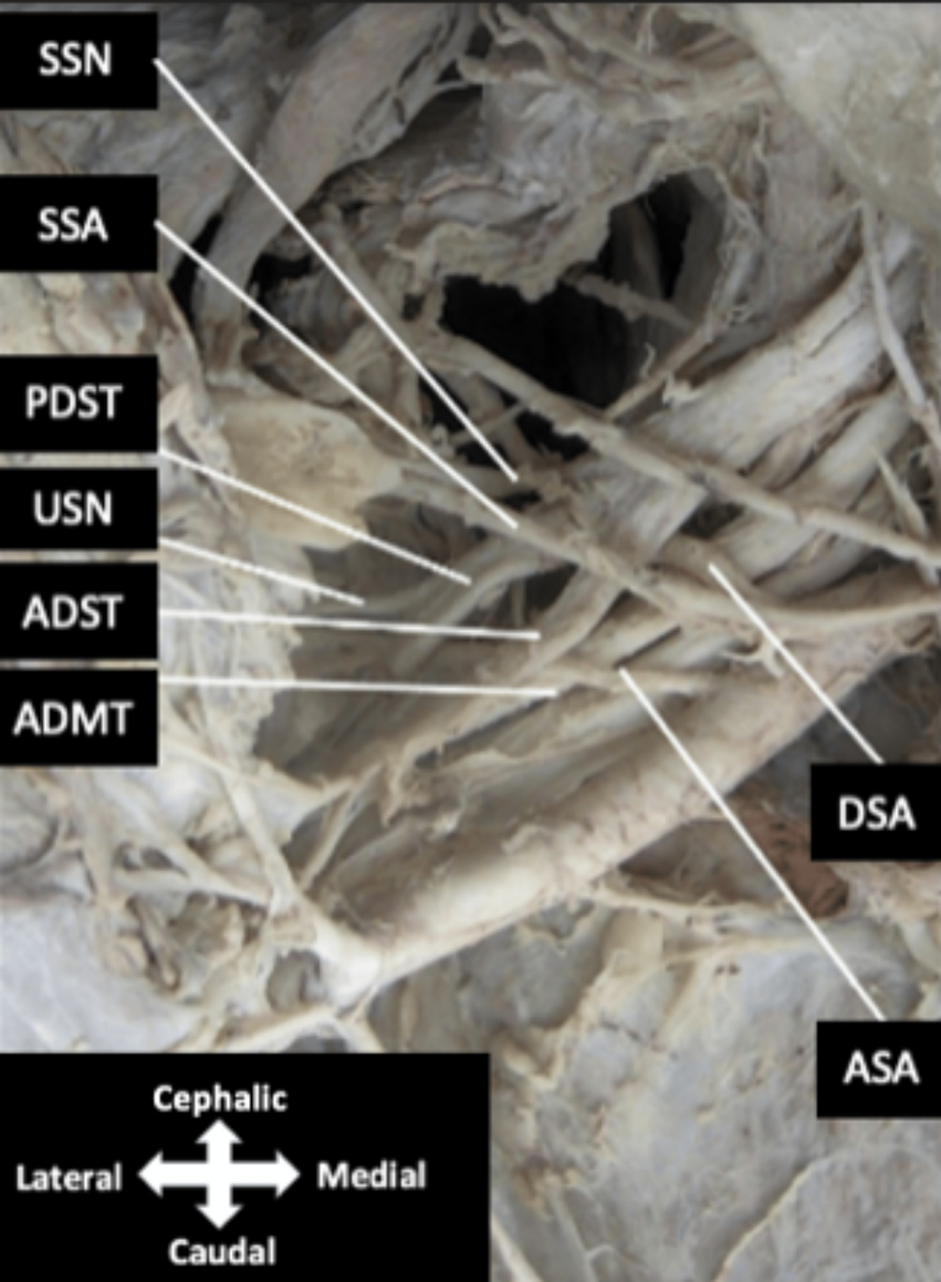
Cervicoaxillary canal The posterior division of the superior trunk is observed giving off the upper subscapular nerve. The accessory subscapular artery can be seen traveling between the anterior division of the superior and middle trunks before traveling with the upper subscapular nerve. The suprascapular nerve and suprascapular artery can be seen traveling together as expected. ADMT, anterior division of middle trunk; ADST, anterior division of superior trunk; ASA, accessory subscapular artery; DSA, dorsal scapular artery; PDST, posterior division of superior trunk; SSA suprascapular artery; SSN, suprascapular nerve; USN, upper subscapular nerve.

Upon dissection of the root of the neck, two arteries were observed branching from the third part of the right subclavian artery before coursing between components of the brachial plexus. The dorsal scapular artery meandered between the superior and middle trunks of the brachial plexus on its path to supply the levator scapulae and rhomboid muscles. The other branch, identified as an accessory subscapular artery, emerged from the subclavian artery 0.8 cm lateral to the dorsal scapular artery, though medial to the first rib, before traveling between the anterior divisions of the superior and middle trunks (Figures 1, 2).

**Figure 3:**
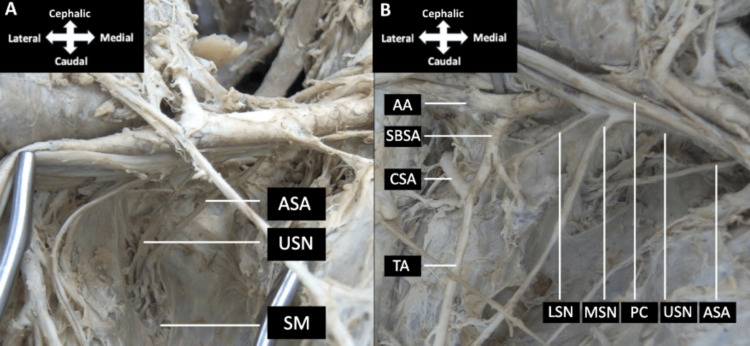
The axilla Dissection of the anterior aspect of the subscapularis muscle (A) revealed the upper subscapular nerve and accessory subscapular artery traveling to supply the subscapularis muscle belly. Further dissection of the axilla (B) revealed an axillary artery giving off the subscapular artery, which branched into the circumflex scapular and thoracodorsal arteries. Dissection of the posterior cord revealed branching of middle and lower subscapular nerves. AA, axillary artery; ASA, accessory subscapular artery; CSA, circumflex scapular artery; LSN, lower subscapular nerve; MSN, middle subscapular nerve; PC, posterior cord; SBSA, subscapular artery; SM, subscapularis muscle; TA, thoracodorsal artery; USN, upper subscapular nerve.

This accessory subscapular artery was present in addition to the subscapular artery, posterior circumflex humeral artery, suprascapular artery, and dorsal scapular artery, all of which demonstrated typical branching patterns to supply the rotator cuff. Further dissection of the accessory subscapular artery revealed that it traveled with the upper subscapular nerve after emerging from the posterior division of the superior trunk of the brachial plexus (Figure 3A). This artery and nerve traveled caudally on the anterior aspect of the subscapularis muscle before piercing into the upper portion of the muscle (Figure 3B). The remainder of the dissection of the brachial plexus revealed typical branching.

## Discussion

Anatomical variations of the brachial plexus in human infant and adult cadavers are well documented. Kerr lists 29 different forms of the brachial plexus among 175 cadaver specimens dissected between 1895 and 1910 [9]. Variations of the lateral cord that involve the musculocutaneous and median nerves are the most common of all reported brachial plexus variations [10]. It has also been reported in the literature that the C4 or T2 spinal nerves may contribute to the plexus [11,12]. There are no reports in the literature of any cases concurrently presenting with a variant upper subscapular nerve and corresponding artery. However, in a cadaveric study exploring variations in the branching of the brachial plexus, Ballesteros and Ramirez (2007) found the origin of the upper subscapular nerve presented a broad range of variability. Of the 57 upper subscapular nerves they evaluated, 50% originated from the posterior division of the superior trunk, though there was no indication of arterial variations coexisting with upper subscapular nerves arising from the posterior division of the superior trunk [13].

In another case report by Deshmukh et al. (2016), the upper subscapular nerve was reported to originate in a triplet fashion resulting in accessory upper subscapular nerves. Of these accessory nerves, one originated directly from the posterior cord, the other from the lower subscapular nerve, and the remaining third midway between the other two [14]. Emamhadi et al. (2016) observed 64 brachial plexuses and found, in one case, the origin of an upper subscapular nerve to arise from the superior trunk of the brachial plexus [15]. Yet another case reported a unique variation in which the posterior cord was replaced by the posterior division of the upper trunk and gave rise to the upper subscapular nerve and axillary nerve, and continued as the radial nerve [16]. None of these studies, or cases, describe any arterial variation coexisting with the aberrant branching patterns of the upper subscapular nerve.

The presence of multiple branches from the third part of the subclavian artery is a unique finding by itself. The dorsal scapular artery has been found to branch from the third part of the subclavian artery 67-75% of the time with the thyrocervical trunk being the second most common origin [6]. However, Ikka et al. found the dorsal scapular artery to originate from the subclavian artery about 61% of the time [17]. As the dorsal scapular artery travels within the supraclavicular triangle, the vessel typically meanders between the superior and middle trunks of the brachial plexus on its path to supply the levator scapulae and rhomboid muscles. This pathway can cause difficulty in placing a supraclavicular brachial plexus block as described by Kinjo and Frankel [18]. An additional vessel accompanying the dorsal scapular artery and meandering through the brachial plexus could serve to increase the difficulty of placing a supraclavicular block and the risk of damage to the blood vessel.

The literature is ripe with reports of arterial branching pattern variations supplying the rotator cuff muscles. For example, the subscapular artery typically arises from the third part of the axillary artery; however, it has also been reported to arise from the second part of the axillary artery or be completely absent with thoracodorsal and circumflex scapular arteries arising separately from the axillary artery [19]. The suprascapular artery typically arises from the thyrocervical trunk, but the vessel can also emerge from the third part of the subclavian artery, different parts of the axillary artery, or the internal thoracic artery [20-22]. The origins of the posterior circumflex humeral artery can also vary as it can arise from a common trunk with subscapular or anterior humeral circumflex arteries or branch from the deep brachial artery [23].

Explanations for these variations in branching from the subclavian artery and the subsequent relationship of those branches with the brachial plexus may be the result of embryologic developmental differences [24]. An initial capillary plexus originating from the dorsal aorta enters the limb bud when it begins its outgrowth. This vascular plexus develops at the same rate as the limb and begins a maturation process involving enlargement and differentiation. It is suggested that the persistence, enlargement, and differentiation of these capillaries, which would normally remain in a capillary state or even regress, give rise to arterial variations [25].

Knowledge of this atypical relationship between the upper subscapular nerve and the accessory subscapular artery is important for anatomists, radiologists, surgeons, and anesthesiologists due to its clinical and surgical implications. Neurovascular variations must be kept in mind whenever surgical access is needed to repair vascular or neurological lesions in the axilla, scapular region, or posterior triangle. The location of this anomalous artery could also create difficulty in placing a supraclavicular block because it increases the risk of damage to the blood vessel. Atypical relationships between nerves and arteries could increase the chances of impingement of vessels leading to ischemia and loss of function [6].

## Conclusions

During routine dissection of the root of the neck, a unique variation involving an accessory subscapular artery from the third part of the subclavian was observed. After reviewing the literature, we found this to be a novel variation as there have been no reported cases of this anomaly. As this unique vessel passed between the anterior divisions of the superior and middle trunks of the brachial plexus, it was accompanied by an unusual branch from the posterior division of the superior trunk as it traveled to the subscapularis muscle. To the best of our knowledge, there have been no documented cases of the coexistence of these neurovascular variations. This cadaveric study improves the knowledge of variations in the anatomy of the brachial plexus and branches of the subclavian artery, which is significant to anatomists, radiologists, anesthesiologists, and surgeons.
